# Network analysis of negative emotions in patients with episodic migraine: need for a multidisciplinary perspective

**DOI:** 10.3389/fneur.2024.1418188

**Published:** 2024-07-02

**Authors:** Federica Guerra, Dina Di Giacomo, Jessica Ranieri, Gennaro Saporito, Patrizia Sucapane, Rocco Totaro, Francesca Pistoia

**Affiliations:** ^1^Postgraduate School on Clinical Psychology, University of L’Aquila, L’Aquila, Italy; ^2^Department of MESVA, University of L’Aquila, L’Aquila, Italy; ^3^Department of Biotechnological and Applied Clinical Sciences, University of L’Aquila, L’Aquila, Italy; ^4^San Salvatore Hospital, L’Aquila, Italy

**Keywords:** migraine, health awareness, emotional dimensions, clinical psychology, wellbeing, rehabilitation, cognitive treatment

## Abstract

**Background:**

Episodic migraine (EM) is the second most prevalent neurological disorder worldwide and is responsible for more disability than all other neurological disorders combined. Triggers for the development of migraine include, stress, emotional burden, low blood sugar levels, tobacco, skipped meals, anxious and depressive feelings. Migraine affects both children and adults, occurring three times more frequently in women than in men.

**Objective:**

The aim of this study was to evaluate the psychological profile of EM patients and the relationship among negative emotions in EM patients, analyzing self-efficacy measures in pain management.

**Design:**

We performed an observational study in 60 outpatients aged 18–55 years (mean age 33.8; SD ±10.4) with EM.

**Methods:**

All patients have been enrolled at the Headache Center of the San Salvatore Hospital of L’Aquila. The assessment comprised five standardized psychological self-assessments investigating relevant emotional dimensions and pain self-efficacy, along with two questionnaires assessing migraine-related disability. A network analysis of negative emotions was performed to evaluate which emotional traits and relationships play a crucial role in pain coping and management.

**Results:**

Our findings indicate that migraine significantly impairs the quality of life of patients in their daily lives. Over half of the patients reported experiencing severe disability, with negative emotions significantly influencing their ability to cope with pain and maintain productivity during migraine attacks. Dysphoric variables (irritability, interpersonal resentment, and surrender) were correlated with difficulties in emotion regulation ability and with the capacity of engaging in goal-directed behaviors despite experiencing pain. The ability to regulate one’s emotions and manage dysphoria were positively correlated with pain self-efficacy, whereas positive mental health was associated with individuals’ confidence in performing activities despite experiencing pain.

**Conclusion:**

Negative emotions had a negative correlation with positive mental health and were linked to a lower capacity to carry out daily activities despite experiencing migraine pain. This suggests that psychological interventions could improve mental health and potentially surpassing the effects of pharmacological interventions alone in migraine management. An integrated, patient-centered approach may represent an effective paradigm to address and reduce the burden of migraine, leading to a reduction in healthcare costs.

## Introduction

Migraine is a neurological disorder that affects nearly 14% of the population, causing debilitating symptoms (1). Episodic migraine (EM), the most common migraine type, is characterized by the presence of less than 15 days per month with migraine symptoms (2). Patients with migraine often describe pain as initially starting as pressure at the height of the forehead but then progressing deeper into the head. Most commonly, it is characterized as pulsating, throbbing, stinging, stabbing, burning, cutting, and oppressive (3). Psychological impairment is largely associated with the EM condition, with patients frequently reporting symptoms such as insomnia, fatigue, depression, and anxiety (4–8). The association between a higher number of headache days and increased rates of anxiety, depression, and insomnia has been reported (5). Additionally, a higher number of headache days has been linked to an enhanced risk of developing psychosocial impairments (6). A migraine attack is considered much more complex than just a mere experience of pain (3). Pain management consists of a chain of behaviors that can be analyzed using three variables: (a) physical sensations, (b) automatic reactions, and (c) actions according to the interpretation (3). Physical sensations may be driven by pain localization (e.g., central or in the middle of the head), pain quality (e.g., pulsating, throbbing, stinging, stabbing, burning, cutting, and oppressive), and intensity (ranging from mild to severe). Automatic reactions involve a loss of control, often triggered by the presence of aura symptoms, which are frequently described as more unpleasant than the pain itself (3). Specifically, in the visual aura, phenomena such as flickers of light, little moons, or lightning that gradually increase in size and move further away into the periphery, have been described as disturbing and destabilizing experiences, conveying feelings of loss of control, insecurity, and fear of severe migraine attacks (3). Acts according to the pain interpretation encompass all actions adopted by patients to manage pain, including taking medications, eating something, finding a distraction, waiting out the pain, and attempting to sleep (3). So, the overall experience of each migraine attack is defined by the combination of physical sensations, automatic reactions, and conscious behaviors. The treatment of EM is mainly based on pharmacological interventions, especially now that new drugs with innovative mechanisms of action are available, such as symptomatic treatments (gepants and ditans) and preventive treatments (monoclonal antibodies targeting the calcitonin gene receptor peptide). Despite this, unmet treatment needs remain a reality in patients’ lives (9). A combination of pharmacological and non-pharmacological approaches has been shown to be more effective than either approach alone in achieving positive outcomes and improving treatment adherence (10–12). Tailored pharmacological treatments combined with a multidimensional, patient-centered, behavioral treatment may reduce the burden of migraine in a biopsychosocial perspective (13). Integrated pharmacological and non-pharmacological interventions can reduce medication overuse in acute management of primary headaches (14). Therefore, it is valuable to identify triggers that may exacerbate a predisposition to migraines and to train patients in effective management techniques. Lifestyle and daily living factors, such as stressful events, an unhealthy diet, poor sleep, and lack of exercise, may trigger migraine attacks. Frequent exposure to such factors may also contribute to the progression of migraine disease and its chronicization. Similarly, specific psychological traits, even in the absence of clear psychiatric comorbidities, may contribute to the worsening of migraine frequency and severity (4). The main psychological interventions used in treating migraine include relaxation training (RT), cognitive-behavioral therapy (CBT), and biofeedback (BF) (15). Some studies demonstrated a broad range of efficacy for non-pharmacological interventions, ranging from 20 to 67%. Importantly, there is no evidence to indicate that one approach—whether CBT, RT or BF—is superior to the others. The efficacy of these interventions needs to be more thoroughly defined, as the evidence base still lacks in quality. The methodological weakness of evidence-based non-pharmacological experimental protocols lies in the absence of quantitative clinical trials. Identifying the key elements of emotional regulation in migraine patients may aid in enhancing their management through a combination of pharmacological and non-pharmacological strategies. This may pave the way for promoting education of healthcare professionals toward a biopsychological approach, ultimately leading to improvements in comprehensive headache care pathways.

Therefore, the aims of our study was to analyze the network of active negative emotions in patients with EM and to evaluate how psychological factors and behaviors interact with each other, potentially contributing to the worsening of migraine symptoms and disability.

## Methods

### Participants

Patients consecutively referring to the Headache Center of the S. Salvatore Hospital of L’Aquila in a 6-months period with a diagnosis of migraine were screened for the inclusion in the study. Migraine diagnosis was performed according to the International Classification of Headache Disorders (ICHD) criteria 3rd edition (2), by a neurologist with expertise in headache diagnosis and management. Inclusion criteria were: (i) age > 18 years, (ii) diagnosis of episodic migraine with or without aura, and (iii) availability to participate in the study and to sign the informed consent form. Exclusion criteria were (a) previous or ongoing history of psychiatric diseases based on ICD-10 classification (b) treatment with antidepressants/mood stabilizers at the time of the study. The presence of these criteria was investigated by asking the patient if there was any previous or current diagnosis of psychiatric diseases and by consulting the digital database of the patient’s previous visits available in our clinics.

Ethical approval to conduct this study was granted by the Institutional Review Board (IRB) of the University of L’Aquila, Italy (ID 29/2023). Informed consent was obtained from each participant, and the study adhered to guidelines outlined in the Declaration of Helsinki (16).

### Measures

Two types of participant information were collected. First, demographics were collected through participant self-reports. We selected independent variables to include in the analysis as they were age/stage of life characteristics (e.g., employment status, marital status, and educational level) related to time from diagnosis. Second, clinical data were obtained from the participants’ medical records, including current stage of disease and the type of medical (pharmacological/surgery) treatment received. The measurement was based on headache and psychological assessments. The headache measure were Migraine Disability Assessment Score Questionnaire (MIDAS) and Headache Impact Test-6 (HIT-6). Psychological measures were: Pain Self-Efficacy Questionnaire (PSEQ), Nepean Dysphoria Scale (NDS-I), Difficulties in Emotion Regulation Scale-Short Form 18 (DERS), Positive Mental Health Scale (PMH).

#### Migraine assessment

The migraine assessment was conducted using two self-assessment measures to evaluate the impact of migraine: the *Migraine Disability Assessment Score Questionnaire* (MIDAS) (17) and the *Headache Impact Test-6* (HIT-6) (18). MIDAS is a questionnaire that measure headache-related disability over a 3-month period in patients with migraine. It is composed of 7 questions including frequency of headaches and pain. The MIDAS score is based on five disability questions in three dimensions (school or work, household and social functioning). The MIDAS score was derived as the sum of lost days due to headache as follows: one question about the extent to which headaches interfere with nonwork activity (miss-leisure) and two questions each about work (miss work + work-half) and work at home (miss-chore + chore half). The total MIDAS score can be further used to define four grades of headache related disability, including grade I for “minimal or infrequent disability” (0–5); grade II for “mild or infrequent disability” (6–10); grade III for “moderate disability” (11–20); and grade IV for “severe disability” (>21). The Cronbach’s alpha was 0.83.

The HIT-6 is a self-report that measures the impact of headache. It comprises six items that assess the adverse impact of headaches on social functioning, role functioning, vitality, cognitive functioning, and psychological distress (18). Headache impact severity level can be categorized using four headache impact severity categories: (1) little or no impact (49 or less), (2) some impact (50–55), (3) substantial impact (56–59), and (4) severe impact (60–78).

#### Psychological assessment

A comprehensive psychological battery including standardized self-assessments tools measuring emotional traits (depression, anxiety, stress, and psychological distress), self-efficacy skills, emotional regulation, and personality dimensions, was used. The participants completed the tests following the individual clinical interview session. Each standardized test was applied using the Italian population version. Specifically, the battery included the following tests:

- The *Depression Anxiety Stress Scales 21* (DASS-21) (19) that is a self-administered questionnaire measuring the negative emotion traits and the degree of severity of the core symptoms of depression, anxiety, and stress. It is composed of 21 questions with responses on a four-point Likert-type scale;
- The *Pain Self-Efficacy Questionnaire* (PSEQ) (20) that is a self-report questionnaire measuring the confidence of individuals experiencing ongoing pain in performing activities despite being in pain. It is composed of 10 items with responses on a 6-point Likert-type.
- The *Nepean Dysphoria Scale* (NDS) (21), a questionnaire that measures dysphoria through the following four subscales: irritability, discontent, surrender and interpersonal resentment. It is composed of 24 items with responses on a 4-point Likert-type. The reliability of test was Cronbach’s α > 91.
- The *Difficulties in Emotion Regulation Scale- Short Form 18* (DERS) (22), a test assessing individual differences in the ability to identify, accept and manage emotional experiences; the test is composed of 6 indexes: (a) Non acceptance (=lack of acceptance of one’s emotions), (b) Goals (=lack of ability to engage in goal-directed activities during negative emotions), (c) Impulse (=lack of ability to manage one’s impulses during negative emotions), (d) Awareness (=lack of awareness of one’s emotions), (e) Strategies (=lack of access to effective emotion regulation strategies), (f) Clarity (=lack of clarity about the nature of one’s emotions).
- The *Positive Mental Health Scale* (PMH) (23), a questionnaire measuring positive mental health, mainly emotional, but also psychological and social aspects of wellbeing. It is composed of 9 items with responses on a 4-point Likert-type. People who are mentally healthy tend to have stable relationships, view their lives as having purpose and direction, experience more positive affect, and are more likely to be self-accepting.

### Study design, procedures, and study flow

This was an observational prospective study investigating patients consecutively referring to the Headache Center. The Medical staff in the Headache Center identified eligible patients. Informed consent was obtained at the time of enrolment. During the first visit medical staff collected clinical data, whereas trained clinical psychologists (blinded to the study’s objectives) performed the psychological assessment in a dedicated room. The psychological evaluations lasted 15 min, and the data was managed anonymously.

### Statistical analysis

Descriptive statistics (mean, standard deviation, percentage) were performed: continuous variables were expressed as the mean ± standard deviation, while categorical variables were presented as frequency or percentage Partial correlation analysis was conducted to examine the relationship between all variables. The Jamovi stat was applied for statistical analyses. The level of significance adopted was α < 0.05. Then, R4.1.1 software was used to process the network analysis. The tuning parameter of EBIC was set to 0.5 and the Pearson correlation method was used. In the network model, edges represent the net correlations between two nodes after statistical control of interference from other nodes in the network.

## Results

### Participants

Sixty-nine patients were considered eligible and invited to participate in the study. Out of them, 60 outpatients aged 18–55 (mean ± SD 33.8 ± 10.4) were finally included as available to participate and providing a signed informed consent. Women (*n* = 50, mean age ± SD 33.8 ± 10.6) were more represented than men (n = 10 mean age ± SD 33.9 ± 9.6). Migraine without aura was the most common diagnosis (68.4%). The mean number of monthly migraine headache days (MHDs) was 5.6 ± 1.8. On the MIDAS assessment, most patients reported severe disability (52%), while the remainder reported moderate (15%), minimal (23%), or mild (10%) disability. The HIT-6 test revealed a high negative impact of migraine on daily life: 73% of patients reported an extremely severe impact, 7% reported a severe impact, and 20% reported a moderate impact. All demographic characteristics of the participants are reported in Table 1. The raw scores, including mean values and standard deviations, obtained on the psychological testing battery are reported in Table 2.

**Table 1:** Demographic characteristics of the participants.

	EM sample (*N* = 60)
Demographics	
Age (years)	33.8 SD ± 10.4
Body mass index (BMI)	23.8 SD ± 3.31
Gender: n (%)	
Male	10 (16.6%)
Female	50 (83.4%)
Marital status: n (%)	
Single	24 (40.0%)
Married	36 (60.0%)
Educational level: n (%)	
Graduate	36 (60.0%)
No graduate	24 (40.0%)
Occupational status: n (%)	
Unemployed	4 (7.0%)
Employed	29 (48.0%)
Self-employed	11 (18.0%)
Student	16 (27.0%)
Smoking habits: n (%)	
Yes	24 (40.0%)
No	36 (60.0%)
Physical activity: n (%)	
Yes	24 (40.0%)
No	36 (60.0%)
Headache characteristics	
Type Migraine: n (%)	
With Aura	19 (32.0%)
Without Aura	41 (68.0%)
MHDs	5.6 ± 1.8
Headache intensity (0–10)	8.16 ± 1.68
MIDAS grade 1/2/3/4 (%)	10/23/15/52
Total MIDAS score	28.43 ± 25.52

**Table 2:** Performances of participants to the standardized tests.

			Shapiro–Wilk	
	Mean	SD	*W*	*p*
Headache measure				
MIDAS	28.43	25.53	0.901	0.001
HIT-6	62.92	6.52	0.967	0.109
Psychological measure				
PSEQ	24.22	14.93	0.964	0.072
DASS-21				
Depression	10.77	8.42	0.926	0.001
Anxiety	9.70	7.92	0.899	0.001
Stress	10.77	8.42	0.926	0.001
NDS				
Irritability	6.43	5.09	0.890	0.001
Discontent	5.20	3.95	0.875	0.001
Interpersonal resentment	3.68	3.75	0.854	0.001
Surrender	3.90	4.05	0.866	0.001
DERS				
Awareness	7.98	3.16	0.950	0.016
Clarity	6.13	2.91	0.887	0.001
Goals	6.05	2.42	0.921	0.001
Impulse	5.68	2.40	0.892	0.001
Non-acceptance	6.15	2.35	0.897	0.001
Strategies	5.18	2.05	0.866	0.001
PMH	17.58	5.63	0.970	0.148

### Network structure

**Figure 1:**
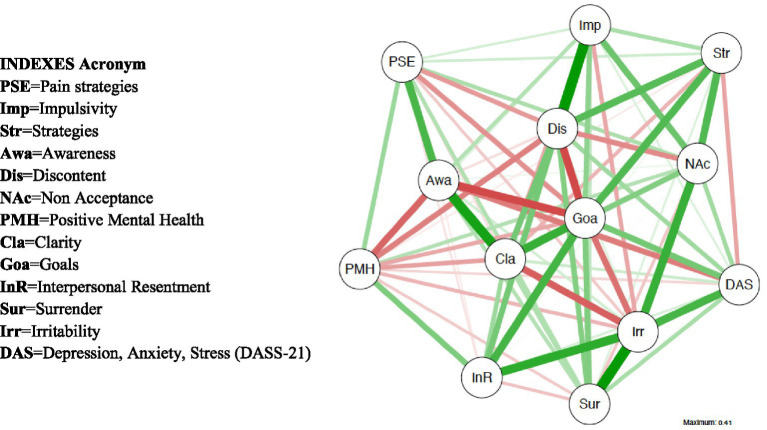
Network structure of negative emotions in EM patients. The green and red edges represent positive and negative partial correlations among nodes. The thick edges and saturated color represent a strong correlation: nodes are the variables (in this case, emotions), and edges are the relationships between the variables.

**Figure 2:**
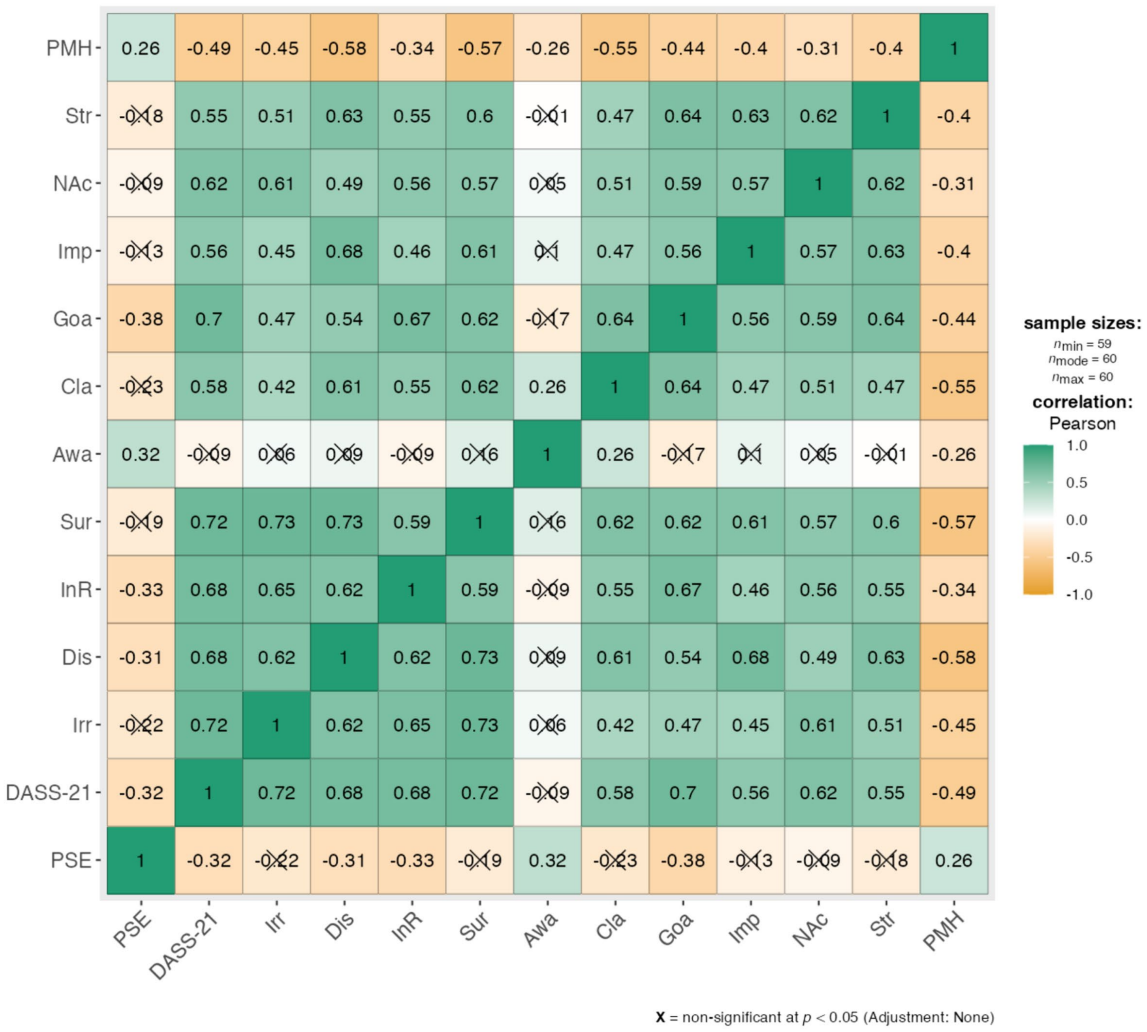
Chart representation of correlation matrix (PMH, Positive Mental Health; Str, strategies; NAc, Non Acceptance; Imp, Impulsivity; Goa, Goals; Cla, Clarity; Awa, Awareness; Sur, Surrender; Irr, Irritability; DASS-21, Depression, Anxiety, Stress; PSE, Pain strategies).

The network structure of different components of negative emotions is shown in Figure 1. We investigated 40 edges across negative emotions, of which 24 were positive and 16 negative. In the cross-community edges, all NDS indexes were positively correlated with DERS indexes: Irritability [Clarity (*r* = 0.001), Goals (*r* = 0.001), Impulsivity (*r* = 0.001), Non acceptance (*r* = 0.001) and Strategies (*r* = 0.001)]; Discontent: [Clarity (*r* = 0.001), Goals (*r* = 0.001), Impulsivity (*r* = 0.001), Non acceptance (*r* = 0.001) and Strategies (*r* = 0.001)]; Interpersonal resentment: [Clarity (*r* = 0.001), Goals (*r* = 0.001), Impulsivity (*r* = 0.001), Non acceptance (*r* = 0.001) and Strategies (*r* = 0.001)]; Surrender: [Clarity (*r* = 0.001), Goals (*r* = 0.001), Impulsivity (*r* = 0.001), Non acceptance (*r* = 0.001) and Strategies (*r* = 0.001)]. Even, NDS indexes were positively correlated with negative traits of behaviors (DASS-21): [Clarity (*r* = 0.001), Goals (*r* = 0.001), Impulsivity (*r* = 0.001), Non acceptance (*r* = 0.001) and Strategies (*r* = 0.001)]. Then, NDS and DERS indexes were negatively correlated with positive mental health value (PMH; *r* = 0.001). Pain self-efficacy value (PSE) correlated with Discontent and Interpersonal resentment (NDS indexes; respectively *r* = 0.01, *r* = 0.01); Goal (DERS index; *r* = 0.002); on contrary, it was correlated positively with PMH (*r* = 0.04). Finally, negative psychological dimensions (DASS-21) were correlated positively with all indexes of NDS (*r* = 0.0001), among DERS indexes almost resulted significant (*r* = 0.001; no significance for Awareness variable), and then negatively correlated with PMH (*r* = 0.001) and PSE (*r* = 0.01). The chart representation of correlation matrix is shown in Figure 2. The correlation matrix of the network is displayed in Table 1.

## Discussion

Our results highlighted interesting pathways that could serve as emotional targets for interventions aimed at customizing and improving health management behaviors in migraineurs. The performed network analysis revealed how different emotions are interconnected in patients with EM, showing complex relationships within specific clusters of emotions.

The strongest positive relationships were found among emotional awareness, emotional clarity, pain self-efficacy and ability to maintain goal-directed behaviors during pain: these emotions form a sort of chain, so that one emotion switches into another, thus mutually reinforcing each other. Emotional awareness is the conscious experience of emotions while emotional clarity refers to one’s ability to identify the type of emotions one is experiencing (24). The process of recognizing and regulating emotions can profoundly impact the perception of pain and one’s ability to cope with it. Low emotional awareness and clarity may correlate with maladaptive behaviors in response to pain, resulting in reduced pain self-efficacy. Conversely, high awareness and clarity of one’s emotions typically foster adaptability, leading to greater confidence in performing specific behaviors or tasks despite experiencing pain (25). The dysphoria variables correlated positively with difficulties in emotion regulation ability: the EM patients seemed irritated, discontent, surrendered, feeling interpersonal resentment on depending to (a) the lack of clarity regarding to the nature of one’s emotions, as well, (b) the lack of ability to engage in goal-direct activities during negative emotions, (c) the lack of ability to manage own impulsivity, (d) the lack of ability to acceptance of one’s emotions, and then (e) the lack of awareness of one’s emotions. These negative emotions were correlated negatively with the positive mental health, whereas positive mental health was associated with the confidence that people with ongoing pain have in performing activities while in pain (= pain self-efficacy). Finally, negative psychological traits (cumulative dimensions of depression, anxiety and stress) were associated with all the examined negative emotions and dimensions, except with the lack of one’s emotions awareness. Dysphoria is characterized by a dynamic state of intense discontent and unhappiness, associated with feelings of inner tension, often accompanied by a tendency to give up or an urge to resort to some action to alleviate such discontent or unhappiness (21). In patients with EM, dysphoric variables are associated with the capacity to engage in goal-directed behaviors despite experiencing pain, as well as with emotional acceptance and access to emotion regulation strategies. Specifically, patients showed irritability, discontent, surrender, and interpersonal resentment associated with a lack of clarity regarding the nature of one’s emotions, an inability to engage in goal-directed activities during negative emotions, difficulty in managing impulsivity, a lack of acceptance of one’s emotions and a lack of awareness of one’s emotions. All negative emotions were negatively correlated with positive mental health, while positive mental health was associated with the confidence that patients with ongoing pain have in performing activities despite being in pain (pain self-efficacy).

Our finding highlighted the psychological dynamics of EM patients coping with headache: they experience a multitude of emotions concurrently during pain, with difficulties in recognizing which emotion is experienced first. In this context, our study specifically investigated the network structure of negative emotions and highlighted that complex psychological dynamics might be active in EM patients dealing with headache: they experience a lack of wellness due to integrated physical and mental symptoms (headaches and negative emotions), ultimately resulting in poor health management. Psychological factors play a crucial role in influencing the perception of pain and patients’ ability to manage it, maintaining good autonomy and productivity even during migraine attacks. Our findings are consistent with previous research, underscoring the severe impact of migraine on patients’ daily quality of life, with depression, anxiety, stress, and sleep disorders exacerbating migraine and reducing wellbeing (4, 6, 8, 26–30). Overall, this suggests that the clinical assessment of patients with migraine should be more comprehensive, encompassing the evaluation of patients’ emotional awareness and the correlation between their emotions and pain. In fact, pain sensitization is highly influenced by psychosocial factors, so that psychological interventions and emotional processing treatments may have a chance in reducing pain severity and improving pain-related disability. This aspect should be taken into consideration also in the process of development of reliable tools and patient-reported outcome measures to assess headache-related disability: in fact, it is current opinion that the scales currently used (for instance MIDAS and HIT 6) have some limitations, mainly represented by the poor correspondence between the dimensions investigated at the patient level and the drivers of reduced health, as expressed at population level, through disability weights (DW) and years lived with a disability (YLDs) developed by the Global Burden of Disease Study (GBD) (28). Our findings reveal that neglecting either integrated physical or emotional factors fails to capture the entire experience of headache disability, thereby interfering with adequate health management. Although we are in a revolutionary period for migraine therapy, with the availability of innovative treatments that differ from the previous ones due to a more specific mechanism of action, there is always a proportion of patients who do not fully improve, do not improve sufficiently, or are refractory to these treatments. In these patients, the presence of specific psychological factors or a maladaptive tendency to manage their own emotions could be the cause of the inadequate success of pharmacological treatments. Therefore, the use of non-pharmacological treatments, not to replace pharmacological ones, but to be used in a complementary manner, could be the missing piece to help all patients and reduce migraine-related disability, which is a significant source of both direct and indirect costs for society. The most recent literature review highlighted the efficacy range of Cognitive Behavioral Therapy (CBT), Relaxation Training (RT), and Biofeedback (BF) as the most commonly applied non-pharmacological interventions, ranging from 20 to 67% (15, 31). According to the biopsychosocial model, pharmacotherapy and behavioral therapy may complement each other, with the most significant reduction in headache frequency achieved by implementing a combination of the two (30).

Strengths of our study include the observational design, the rigorous selection of patients with EM, excluding those with psychiatric comorbidities or therapies, the multidisciplinary assessment performed by both neurologists and psychologists and the adoption of a network analysis model. In particular, the latter is an innovative model of analysis that enables the recognition of patterns of statistical association in multivariate psychological and behavioral data, by identifying system components (network nodes) and the relations among them (links between nodes). This analysis are often carried out with the goal of relating structural features of the network to system dynamics, without requiring strong *a priori* assumptions about associations (32).

Potential limitations of our study include the sample size, which could be expanded in future studies to provide more robust evidence, the inability to establish causal relationships among the investigated variables in the network analysis. In fact, the described relationships are purely statistical associations and causal inference is not justified, as edges between nodes may arise owing to directed causal effects or feedback loops, but also owing to unobserved common causes (33). Last, the study did not provide a follow-up measure regarding the changing frequency, severity and burden of migraine due to pharmacological interventions. Future longitudinal studies will provide follow-up information on the evolving pattern of migraine, in terms of frequency, severity and disability, as well as any associated psychological changes resulting from therapeutic interventions.

In conclusion, more effective psychological interventions for EM patients might focus on the relationship between dysphoria variables and difficulties in emotion regulation ability. Based on these preliminary findings, which need further confirmation through studies with larger sample sizes, a practical suggestion for clinicians may be to adopt an integrated biopsychosocial approach for patient care. This approach should be based on multidisciplinary assessment and management, taking into consideration both the clinical and psychological aspects of the patients. Complementary pharmacological and behavioral treatments, based on personalized medicine to enhance the patient-centered approach, could be the focus of future research protocols. Integrated interventions should be tailored to consider the dynamics of negative emotion onset and consolidation.

This approach could address the unmet needs of patients, improve clinical care, and enhance quality of life.

## Data availability statement

The raw data supporting the conclusions of this article will be made available by the authors, without undue reservation.

## Author contributions

FG: Conceptualization, Data curation, Writing – original draft. DG: Conceptualization, Writing – review & editing. JR: Formal analysis, Investigation, Writing – original draft. GS: Investigation, Writing – original draft. PS: Methodology, Writing – original draft. RT: Methodology, Writing – original draft. FP: Conceptualization, Data curation, Writing – review & editing.

## References

[1] Stovner LJ Hagen K Linde M Steiner TJ. The global prevalence of headache: an update, with analysis of the influences of methodological factors on prevalence estimates. J Headache Pain. (2022) 23:34. doi: 10.1186/s10194-022-01402-2, PMID: 35410119 PMC9004186

[2] Headache Classification Committee of the International Headache Society (IHS). The International Classification of Headache Disorders, 3rd edition. Cephalalgia. (2018) 38:1–211. doi: 10.1177/0333102417738202 29368949

[3] Persson M Rembeck G Weineland S. Conceptualising migraine attacks from a biopsychosocial model using qualitative and functional behavioural analysis. Scand J Prim Health Care. (2023) 41:257–66. doi: 10.1080/02813432.2023.2231034, PMID: 37409784 PMC10478623

[4] Pistoia F Salfi F Saporito G Ornello R Frattale I D’Aurizio G. Behavioral and psychological factors in individuals with migraine without psychiatric comorbidities. J Headache Pain. (2022) 23:110. doi: 10.1186/s10194-022-01485-x, PMID: 36028795 PMC9411831

[5] Buse DC Reed ML Fanning KM Bostic R Dodick DW Schwedt TJ. Comorbid and co-occurring conditions in migraine and associated risk of increasing headache pain intensity and headache frequency: results of the migraine in America symptoms and treatment (MAST) study. J Headache Pain. (2020) 21:23. doi: 10.1186/s10194-020-1084-y 32122324 PMC7053108

[6] Ruscheweyh R Müller M Blum B Straube A. Correlation of headache frequency and psychosocial impairment in migraine: a cross-sectional study. Headache. (2014) 54:861–71. doi: 10.1111/head.12195 23980919

[7] George A Minen MT. Episodic migraine and psychiatric comorbidity: a narrative review of the literature. Curr Pain Headache Rep. (2023) 27:461–9. doi: 10.1007/s11916-023-01123-4, PMID: 37382869

[8] Lucchesi C Baldacci F Cafalli M Dini E Giampietri L Siciliano G. Fatigue, sleep–wake pattern, depressive and anxiety symptoms and body-mass index: analysis in a sample of episodic and chronic migraine patients. Neurol Sci. (2016) 37:987–9. doi: 10.1007/s10072-016-2505-1, PMID: 26879311

[9] Bentivegna E Galastri S Onan D Martelletti P. Unmet needs in the acute treatment of migraine. Adv Ther. (2024) 41:1–13. doi: 10.1007/s12325-023-02650-7, PMID: 37943442 PMC10796525

[10] Holroyd KA Cottrell CK O'Donnell FJ Cordingley GE Drew JB Carlson BW. Effect of preventive (blocker) treatment, behavioural migraine management, or their combination on outcomes of optimised acute treatment in frequent migraine: randomised controlled trial. BMJ. (2010) 341:c4871–1. doi: 10.1136/bmj.c4871, PMID: 20880898 PMC2947621

[11] Lemstra M Stewart B Olszynski WP. Effectiveness of multidisciplinary intervention in the treatment of migraine: a randomized clinical trial. Headache. (2002) 42:845–54. doi: 10.1046/j.1526-4610.2002.02202.x 12390609

[12] Holroyd KA France JL Cordingley GE Rokicki LA Kvaal SA Lipchik GL. Enhancing the effectiveness of relaxation-thermal biofeedback training with propranolol hydrochloride. J Consult Clin Psychol. (1995) 63:327–30. doi: 10.1037/0022-006X.63.2.327, PMID: 7751496

[13] Freitag FG Lake A Lipton R Cady R US Headache Guidelines Consortium, Section on Inpatient Treatment Chairpersons Diamond S. Inpatient treatment of headache: an evidence-based assessment. Headache. (2004) 44:342–60. doi: 10.1111/j.1526-4610.2004.04093.x, PMID: 15109359

[14] Grazzi L Andrasik F. Medication-overuse headache: description, treatment, and relapse prevention. Curr Sci Inc. (2006) 10:71–7. doi: 10.1007/s11916-006-0012-4, PMID: 16499833

[15] Sullivan A Cousins S Ridsdale L. Psychological interventions for migraine: a systematic review. J Neurol. (2016) 263:2369–77. doi: 10.1007/s00415-016-8126-z, PMID: 27159991 PMC5110589

[16] Goodyear MDE Krleza-Jeric K Lemmens T. The declaration of Helsinki. BMJ. (2007) 335:624–5. doi: 10.1136/bmj.39339.610000.BE, PMID: 17901471 PMC1995496

[17] Stewart WF Lipton RB Dowson AJ Sawyer J. Development and testing of the migraine disability assessment (MIDAS) questionnaire to assess headache-related disability. Neurology. (2001) 56:S20–8. doi: 10.1212/WNL.56.suppl_1.S20 11294956

[18] Wang Y-F Wang S-J Huang YH Chen YT Yen YC Shia BC. Treatment pattern and health care resource utilization for Taiwanese patients with migraine: a population-based study. Front Neurol. (2023) 14:1222912. doi: 10.3389/fneur.2023.1222912, PMID: 37654430 PMC10466390

[19] Bottesi G Ghisi M Altoè G Conforti E Melli G Sica C. The Italian version of the depression anxiety stress Scales-21: factor structure and psychometric properties on community and clinical samples. Compr Psychiatry. (2015) 60:170–81. doi: 10.1016/j.comppsych.2015.04.005, PMID: 25933937

[20] Chiarotto A Vanti C Ostelo RW Ferrari S Tedesco G Rocca B. The pain self-efficacy questionnaire: cross-cultural adaptation into Italian and assessment of its measurement properties. Pain Pract. (2015) 15:738–47. doi: 10.1111/papr.12242, PMID: 25264358

[21] D’Agostino A Manganelli E Aportone A Monti MR Starcevic V. Development, cross-cultural adaptation process and preliminary validation of the Italian version of the Nepean dysphoria scale. J Psychopathol. (2016) 30:674. doi: 10.1016/S0924-9338(15)30534-4

[22] Rossi AA Panzeri A Mannarini S. The Italian version of the difficulties in emotion regulation scale – short form (IT-DERS-SF): a two-step validation study. J Psychopathol Behav Assess. (2023) 45:572–90. doi: 10.1007/s10862-022-10006-8

[23] Lukat J Margraf J Lutz R Van Der Veld WM Becker ES. Psychometric properties of the positive mental health scale (PMH-scale). BMC Psychol. (2016) 4:8. doi: 10.1186/s40359-016-0111-x, PMID: 26865173 PMC4748628

[24] Burrowes SAB Goloubeva O Stafford K McArdle PF Goyal M Peterlin BL. Enhanced mindfulness-based stress reduction in episodic migraine—effects on sleep quality, anxiety, stress, and depression: a secondary analysis of a randomized clinical trial. Pain. (2022) 163:436–44. doi: 10.1097/j.pain.0000000000002372, PMID: 34407032 PMC8669060

[25] Fatima I: Health locus of control, illness behavior and headache related quality of life in individuals with migraine. Pain Management (2021).

[26] Moon H-J Seo J-G Park S-P. Perceived stress in patients with migraine: a case-control study. J Headache Pain. (2017) 18:73. doi: 10.1186/s10194-017-0780-8, PMID: 28733942 PMC5520838

[27] Yousefi Afrashteh M Abbasi M Abbasi M. The relationship between meaning of life, perceived social support, spiritual well-being and pain catastrophizing with quality of life in migraine patients: the mediating role of pain self-efficacy. BMC Psychol. (2023) 11:17. doi: 10.1186/s40359-023-01053-1, PMID: 36691101 PMC9869619

[28] Waliszewska-Prosół M Montisano DA Antolak M Bighiani F Cammarota F Cetta I. The impact of primary headaches on disability outcomes: a literature review and meta-analysis to inform future iterations of the global burden of disease study. J Headache Pain. (2024) 25:27. doi: 10.1186/s10194-024-01735-0, PMID: 38433202 PMC10910736

[29] Bolton D. A revitalized biopsychosocial model: core theory, research paradigms, and clinical implications. Psychol Med. (2023) 53:7504–11. doi: 10.1017/S0033291723002660 37681273 PMC10755226

[30] Bolton D. A revitalized biopsychosocial model: core theory, research paradigms, and clinical implications. Psychol Med. (2023) 53:7504–7511. 37681273 10.1017/S0033291723002660 PMC10755226

[31] Pistoia F Sacco S Carolei A. Behavioral therapy for chronic migraine. Curr Pain Headache Rep. (2013) 17:304. 23263845 10.1007/s11916-012-0304-9

[32] Newman MEJ Barabási ALE Watts DJ. The structure and dynamics of networks. Princeton University Press (2006).

[33] Borsboom D Cramer AO. Network analysis: an integrative approach to the structure of psychopathology. Annu Rev Clin Psychol. (2013) 9:91–121. 23537483 10.1146/annurev-clinpsy-050212-185608

